# Controllable Biosynthesis and Properties of Gold Nanoplates Using Yeast Extract

**DOI:** 10.1007/s40820-016-0102-8

**Published:** 2016-09-13

**Authors:** Zhi Yang, Zhaohui Li, Xuxing Lu, Fengjiao He, Xingzhong Zhu, Yujie Ma, Rong He, Feng Gao, Weihai Ni, Yasha Yi

**Affiliations:** 1 Key Laboratory for Thin Film and Micro Fabrication of Ministry of Education, Department of Micro/Nano Electronics, School of Electronic Information and Electrical Engineering, Shanghai Jiao Tong University, Shanghai, 200240 People’s Republic of China; 2 Division of i-Lab, Key Laboratory of Nano-Bio Interface and Collaborative Innovation Center of Suzhou Nano Science and Technology, Suzhou Institute of Nano-Tech and Nano-Bionics, Chinese Academy of Sciences, Suzhou, 215123 Jiangsu People’s Republic of China; 3 Integrated Nano Optoelectronics Laboratory, Department of Electrical and Computer Engineering, University of Michigan, Dearborn, MI 48128 USA; 4 Energy Institute, University of Michigan, Ann Arbor, MI 48109 USA

**Keywords:** Yeast, Gold nanoplates, Biosynthesis, pH dependent, Plasmonic property

## Abstract

**Abstract:**

Biosynthesis of gold nanostructures has drawn increasing concerns because of its green and sustainable synthetic process. However, biosynthesis of gold nanoplates is still a challenge because of the expensive source and difficulties of controllable formation of morphology and size. Herein, one-pot biosynthesis of gold nanoplates is proposed, in which cheap yeast was extracted as a green precursor. The morphologies and sizes of the gold nanostructures can be controlled via varying the pH value of the biomedium. In acid condition, gold nanoplates with side length from 1300 ± 200 to 300 ± 100 nm and height from 18 to 15 nm were obtained by increasing the pH value. Whereas, in neutral or basic condition, only gold nanoflowers and nanoparticles were obtained. It was determined that organic molecules, such as succinic acid, lactic acid, malic acid, and glutathione, which are generated in metabolism process, played important role in the reduction of gold ions. Besides, it was found that the gold nanoplates exhibited plasmonic property with prominent dipole infrared resonance in near-infrared region, indicating their potential in surface plasmon-enhanced applications, such as bioimaging and photothermal therapy.

**Graphical Abstract:**

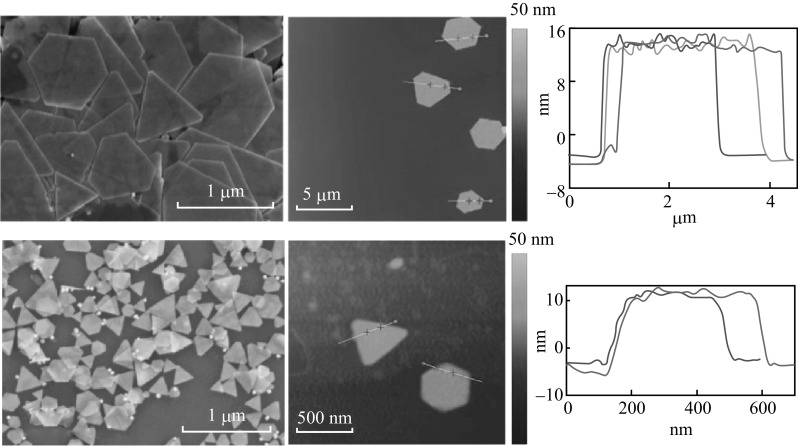

**Electronic supplementary material:**

The online version of this article (doi:10.1007/s40820-016-0102-8) contains supplementary material, which is available to authorized users.

## Introduction

In past decades, gold nanostructures have attracted significant interest due to their potential applications in diverse fields, such as surface-enhanced Raman scattering, photothermal therapy, catalysis, electronics, sensing, and imaging, etc. [1–9]. Since the properties of gold nanostructures can be well controlled by sizes, shapes, and crystal orientations, numerous efforts have been made to fabricate gold nanostructures with various morphologies, including nanoplates, nanowires, nanorods, nanocubes, nanoclusters, etc. [6, 10–13]. Among them, gold nanoplates have attracted increased attention due to their excellent localized surface plasmon resonance properties assigned from their sharp corners and edges. So far, many synthetic strategies were developed to synthesize gold nanoplates, such as seeded-growth method [11, 14, 15], thermal reduction approach [16], electrochemical approach [17], and photocatalytic approach [18]. Notably, Zhang et al. have reported monodispersed triangular gold nanoplates with very high morphological yield (>90 %) using a rapid one-pot seedless growth progress, in which iodide ions not only selectively bind to the Au {111} facets but also selectively remove other less stable shape impurities through oxidative etching by forming triiodide ions, thus facilitating the formation of nuclei with dominant planar structure [19]. However, gold nanoplates synthesized by chemical approaches have serious limitations in biomedical applications due to the toxic surfactants, the complexity, and high cost of reducing agents and surfactants [20–22]. Hence, it is necessary for greener and more accessible synthesis of gold nanoplates with high morphological yield [23–29].

Biosynthesis of gold nanostructures has many advantages such as environment-friendly, energy efficiency, biocompability, and low cost [30, 31]. Till now, several strategies, including microorganisms and plants, have been used for the biosynthesis of gold nanoparticles [31]. Comparing with the method using microorganisms, there exist a primary factor limiting the use of plant species in the biosynthesis of gold nanoparticles, in which excess use of plant species may pose a risk and imbalance to the plant diversity [32]. So far, many microorganisms such as Rhizopus oryzae [32, 33], Neurospora crassa [34], and Trichoderma harzianum [35, 36] have been reported to successfully synthesize gold nanoparticles. The biosynthesis of gold nanoparticles using microorganisms is mainly divided into two approaches: intracellular and extracellular. As to the former, it has been demonstrated that enzymes in the microorganisms can transfer electrons from reductants to gold ions, which results in the reduction of gold ions and formation of gold particles. But because the reduction happens in cell, it is difficult to get gold nanoparticles separately, thus hindering the direct application in next steps. As to the latter, the gold ion is reduced by the metabolites of microorganisms. Thus it is convenient to separate Au products using this approach. But it is sometimes inevitable that gold ions may be uptaken by the cell and bound out of the intracellular membrane, which causes the reduction on the surface of the microorganism. In this case, the separation between gold products and microorganisms is also difficult. Among many microorganisms for biosynthesis of gold particles, yeast has lots of advantages because of its abundance, and more importantly its high yield. Yeasts are easy to handle in laboratory conditions, to synthesize high amount of enzymes and grow rapidly employing simple nutrients [37]. However, methods using yeast to prepare gold nanoparticles also have common issues like other biosynthesis methods, that is, the size and morphology are uncontrollable and nonuniform. This is mainly due to the fact that biomedium is too complex to be fully understood and therefore interferences from invalid metabolites and nutrients can hardly be controlled [38–40].

Herein, metabolites of yeast were used to synthesize gold nanoplates. Various morphologies of gold nanoplates (triangle, truncated triangle, and hexagonal nanoplates) with uniform size were fabricated successfully. The water solubility and evolution of gold nanoplates were explored. Properties of active molecules in the biomedium were measured and a postulated synthesis process of nanoplates in the acid condition is given finally. Extinction spectra and charge distribution profiles of the three types of nanoplates are compared by performing finite-difference time-domain (FDTD) calculations, indicating their potential in surface plasmon-enhanced applications.

## Materials and Methods

### Materials

Instant high-sugar dry yeasts were purchased from AB MAURI. Sucrose, sodium hydroxide (NaOH), and chloroauric acid hydrated (HAuCl_4_·4H_2_O) were purchased from Sino Pharm Chemical Corporation, analytically pure and used without any further purification. The deionized water used was purified by the Simplicity Ultrapure Water Systems (18.2 MΩ cm at 25 °C).

### Biological Solution

Firstly, 7.2 g sucrose and 1 g instant dry yeast were dissolved in 300 mL deionized water. Then the mixed solution was co-incubated in a shaker at 35 °C for 48 h. During this period, the concentration of yeast cells and some metabolites were produced and increased significantly. The final pH value of the culture medium was 3.3. Subsequently, the obtained culture medium was purified by a two-step centrifugation of two 15 min centrifugations at 1200 and 8000 rpm, respectively. At the first low speed step, yeast cells were precipitated and prevented from cell rupture. While at the second high speed step, hypha and cell debris were centrifuged as sedimentation. Then the biomedium will go through half-an-hour’s boiling and the third centrifugation step of 8000 rpm for 15 min. Finally, the biological solution was obtained and would serve as both the reducing agents and the surfactants in the following procedure.

### Synthesis of Gold Nanoplates

Yeast culture medium extract in 10 mL was equally distributed into two conical flasks. One remained the same, while the other was adjusted by NaOH (0.5 M) to the pH value of 4.0. Then the two samples were co-incubated with 500 μL HAuCl_4_ (2.5 mM) in water bath for 5 h at 30 °C. The samples went through centrifugation of 3000 rpm for 10 min and re-dispersed in deionized water alternatively to remove biomass in the reaction solution. The final solutions were kept in the refrigerator for 1 month at 4 °C.

### Characterizations

The morphologies of gold NPs were observed by scanning electron microscopy (SEM, Carl Zeiss Ultra Plus, Germany) and transmission electron microscopy (TEM, JEM-2100HT, Japan). Atomic Force Microscope (AFM) images were acquired using a Multimode Nanoscope V scanning probe microscopy system (Bruker, USA). The commercially available AFM cantilever tips with a force constant of 50 N m^−1^ and resonance vibration frequency of 350 kHz (Bruker, USA) were used. The samples of gold nanoplates were prepared by centrifugalizing the crude solution at 10,000 rpm for 10 min to wash out the organics in the supernatant liquid and then re-dispersed by ultrasonic dispersion in aqueous solution for SEM, TEM, and AFM investigations. FT-IR spectrum was recorded on a VERTEX 70 spectrometer (Bruker, Germany) with DTGS or MCT as detector. Protein test was carried out by Coomassie brilliant blue-stained SDS-PAGE on Mini-protean Tetra (Bio-Rad, American) with a ChemiDoc MP imaging system (Bio-Rad, American). LC-ESI/HRMS was performed on a Waters ACQUITY UPLC system equipped with a binary solvent delivery manager and a sample manager, coupled with a Waters Micromass Q-TOF Premier Mass Spectrometer equipped with an electrospray interface (Waters Corporation, Milford, MA). The column used in LC is Acclaim Trinity P1 column (100 × 2.1 mm, 3 µm) (Thermo Scientific, American). Amino acid analysis (AAA) of biomedium was carried out on an automatic amino acid analyzer L-8900 (Hitachi Ltd., Japan). In the simulation, MEEP 49 a FDTD software package was employed and Drude–Lorentz model was used to represent the dielectric function of bulk Au. The refractive index of the surrounding medium was set as 1.33. A plane wave propagating in the direction perpendicular to the plate was used as the excitation source in the simulation. The mesh grid was set as 3.0 nm in size. Charge distribution profiles were calculated at the top surfaces of the plates and at peak wavelengths of the extinction spectrum.

## Results and Discussion

### Controllable Preparation of Gold Nanoplates at Different pH Conditions

**Fig. 1:**
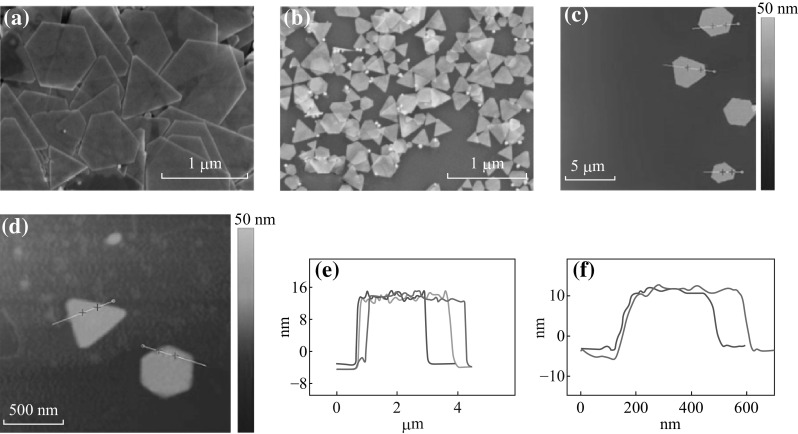
SEM images of **a** gold nanoplates synthesized at low pH without NaOH, and **b** small gold nanoplates synthesized at high pH tuned by NaOH solution. AFM images of: **c** large and **d** small nanoplates. The height profile of **e** large nanoplates, and **f** small nanoplates

In this work yeast was prior cultured in sucrose-only medium at pH 3.3. Figure 1a, b show the SEM images of the gold nanoplates obtained at pH 3.3 and 4.0 condition, respectively. The acid culture solution was obtained using the yeast extract directly without any pre-process, while the basic culture was obtained by adding NaOH solution in the yeast extract. The gold nanoplates prepared at pH = 3.3 show larger sizes with diagonal lengths of 1300 ± 200 nm than those prepared at pH = 4.0 with diagonal lengths of 300 ± 100 nm. As shown in Fig. 1a, b, it can be found that triangle, truncated triangle, and hexagon nanoplates are coexisting. The ratio of triangles is higher in Fig. 1b than those of triangles in Fig. 1a, which is consistent with the report about the transformation between triangles and hexagons [41].

The ratio of triangle and hexagon depends on the stacking faults commonly occurred in close-packed lattices. For the face-centered cubic metal, when a single planar defect (e.g., a twin or a stacking fault) is involved, hexagonal plates can form in the early stage of growth due to the six-fold symmetry of a face-centered cubic lattice. As proposed by previous reports, the presence of a planar defect can cause the six-sided faces, where the defect plane ends, to form alternating concave- and convex-type surfaces [20]. Because each atomic site only has three nearest atomic neighbors on the convex-type surface, the stabilization energy for attaching atoms to this surface is relatively low. As a result, the atoms on this surface tend to be dissolved into solution again, creating a high-energy barrier for the addition of atoms. In contrast, the concave-type surface creates a reentrant groove, a self-perpetuating ledge that increases the number of nearest neighbors for an adatom and thus the stabilization energy. In this case, atomic addition becomes favorable. Taken together, in a crystal with a single planar defect, the fast addition of metal to the concave sides can cause those very faces to grow out of existence, leading to a triangular plate whose side faces are bounded by three convex sides that do not favor atomic addition.

Hexagonal and triangular gold nanoplates often co-exist in the aforementioned polymer or surfactant-assisted process. Only the presence of a single twin plane in the seed is expected to direct growth in two dimensions which limits the final size and pure morphology of the nanoprism, while the presence of two parallel twin planes would make the fast growing edges to regenerate one another, allowing shapes such as hexagonal nanoplates to form [41–43]. Figure 1c, d show the Atomic force microscopy (AFM) images of the two gold nanoplate samples, and the thicknesses of the two kinds of nanoplates are ~18 and ~15 nm, respectively.

**Fig. 2:**
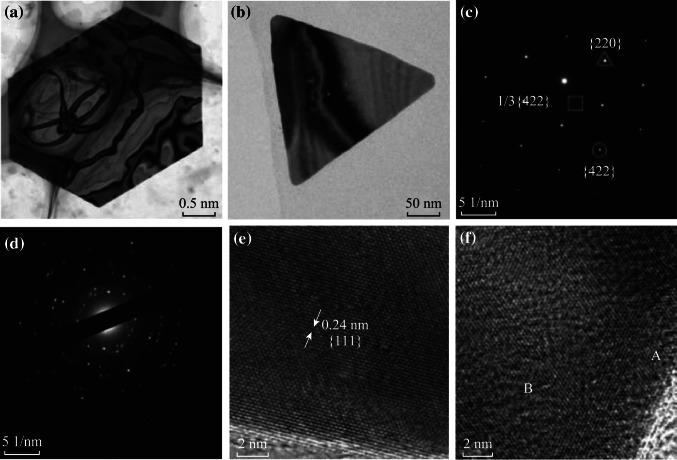
**a**, **c**, **e** corresponding TEM, SAED, and HRTEM images of large gold nanoplates synthesized at low pH without NaOH. **b**, **d**, **f** Corresponding TEM, SAED, and HRTEM images of small gold nanoplates synthesized at high pH tuned by NaOH

TEM and HRTEM images were used to investigate the crystallization of the biosynthesized gold nanoplates, and the results are shown in Fig. 2. The binding contours across the gold nanoplates in Fig. 2a is ascribed to high surface tension [44]. Both the surfaces and the edges in the small gold nanoplates are rougher than the large ones (Fig. 2b). Selected area electron diffraction (SAED) patterns in Fig. 2c and regular crystal lattice in Fig. 2e together demonstrate the well crystallization of the synthesized gold nanoplates. Three sets of characteristic spots marked by square, triangle, and circle in Fig. 2c represent 1/3{422}, {220}, and {422} crystal planes, respectively. The 1/3{422} super lattice pattern occurs when the gold nanoplate contains a {111} twin plane [45]. Figure 2d shows typical patterns of polycrystal, and the corresponding HRTEM image in Fig. 2f shows abundant crystal defections [21, 46, 47]. The regions marked with red arrow A and B reveal the representative fracture and vacancy of the gold nanoplates, respectively. The growth process of the gold nanoplates is displayed in Fig. S1. At the first stage of the reaction, only gold nanoparticles are synthesized. Along with the time, gold nanoplates grow by accumulation and integration of small gold nanoparticles but not completely merge together.

The pH-dependent biosynthesis of gold nanostructures is further explored in the neutral and base condition. When the pH of the biomedium is tuned by NaOH solution, a mixture of nanoplates and nanoflowers, nanoflowers, and small nanoparticles were obtained at the pH values of 5.0, 7.0, and 10.0, respectively (Fig. S2).

### Morphological Evolution of Gold Nanoplates in Aqueous Solution

The morphological evolution was studied in order to observe the stability of the gold nanoplates. The gold products including all the gold nanoplates and nanoparticles were dispersed in deionized water and then kept for a month. It was found that both the gold nanoplates and nanoparticles changed their morphologies, which may be due to the slow recrystallization and rearrangement of gold atoms. According to the Kelvin equation [48], the solid solubility (*C*) in the solution is a function of the solid size, as shown in Eq. 1,

1 $$\ln \left( {\frac{{C_{r} }}{{C_{{r_{0} }} }}} \right) = \frac{2\sigma M}{RT\rho }\frac{1}{r},$$

where *C*_*r*_ and *C*_*r*0_ are the solubility of nanoparticles with diameter *r* and *r*_0_ (*r* > *r*_0_), respectively, *ρ* is the density of gold nanoparticles, *M* is the relative atomic mass, *R* is the ideal gas constant, and *T* is absolute temperature. Based on the above equation, it can be calculated that the equilibrium saturation concentrations of the large-sized nanoplates are much lower than those of the small-sized nanoparticles. After being re-dispersed in deionized water, the saturation equilibrium is further deviated, and the surface protection is weakened. Thus it can be seen that gold nanoplates grow larger to multi-layered ones, as a trade-off between the different equilibrium saturation concentrations. In the same way, gold nanoparticles are gradually etched. The structures of nanoplates would gradually change in order to adapt to the increasing surface tension. As a result, the formation of sub-grains in the nanoplates and the increase of proportion of spiral gold nanoplates will happen in order to release the high surface tension.

**Fig. 3:**
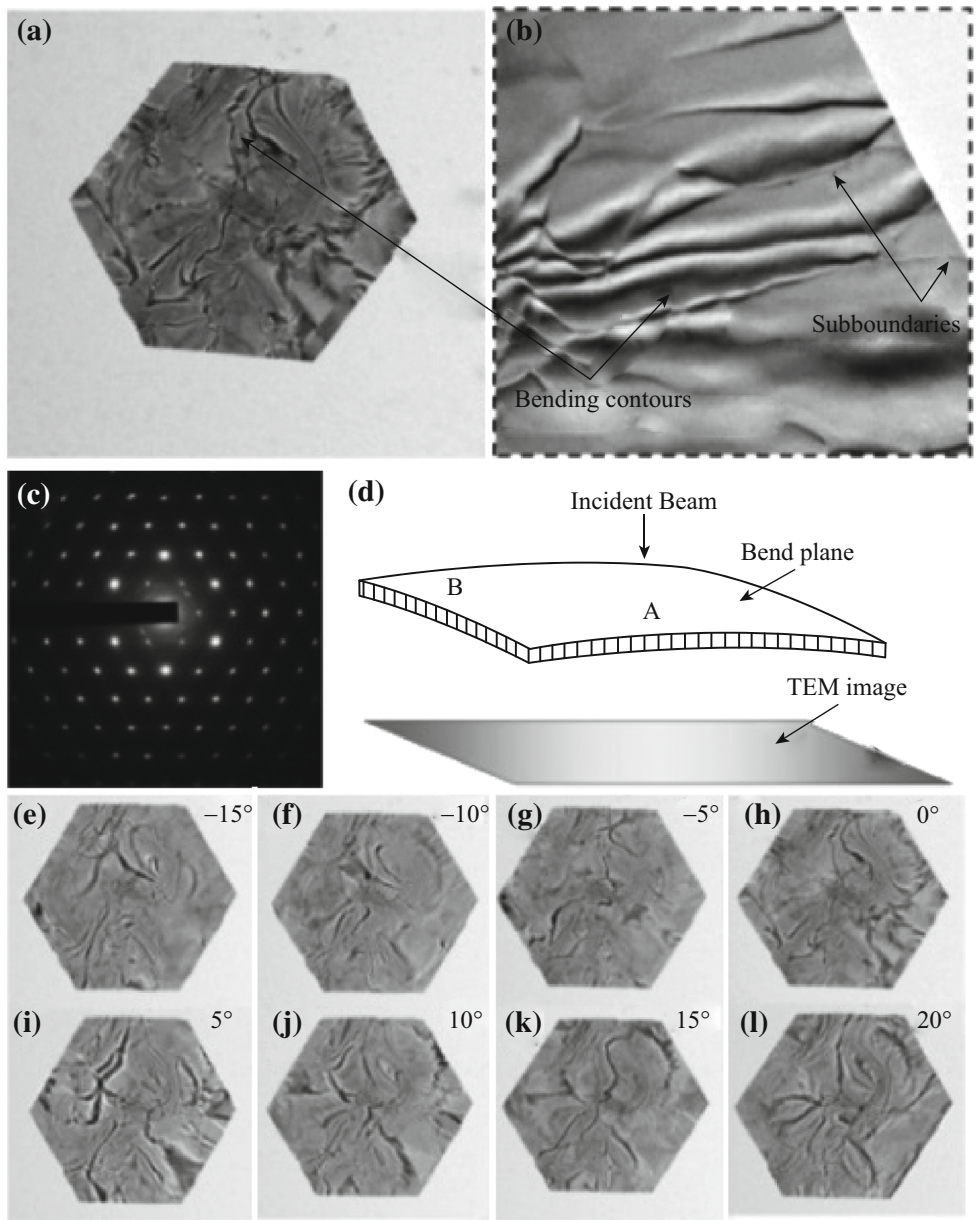
**a** TEM, **b** magnified TEM images, and **c** SAED patterns of the same nanoplate are depicted without tiling the sample holder. **d** Schematic illustration of the contours with small angles. The incident beam is exactly parallel to the crystal planes at *A* and directly transmitted, while the orientation of *B* meets Bragg relationship and diffracted electron beam of *B* tends, resulting in bright contrast in *A* and dark contrast in *B* on the objective aperture. **e**–**l** Bright-field TEM images of the same nanoplate depicted when *X*-axis of the sample holder tiled every 5° from −15° to 20°

The proof of bending contours and sub-grains is confirmed in TEM and HRTEM images (Fig. 3a, b). SAED patterns of the gold nanoplate in Fig. 3c clearly present its structure: (1) the strong 1/3{422} diffraction indicates that there are lots of {111} micro-twinning structures in the nanoplate; (2) the diffraction circles of {111} and {200} reveal the existence of the attached nanoparticles; (3) splitting spots in the circular direction shown in the diffraction patterns (Fig. 3c) reveal diffraction spots of rotated sub-grains. The schematic illustration in Fig. 3d shows how small angles contribute to the contrast of contours. The contrast stripes change their position when the specimen is tilted along the *X*-axis (Fig. 3e–l), also indicating inconsistent lattice orientation in the gold nanoplate. When the lattice in the crystalline structure is hetero-oriented, small angles arise between crystal planes of the same family [49].

**Fig. 4:**
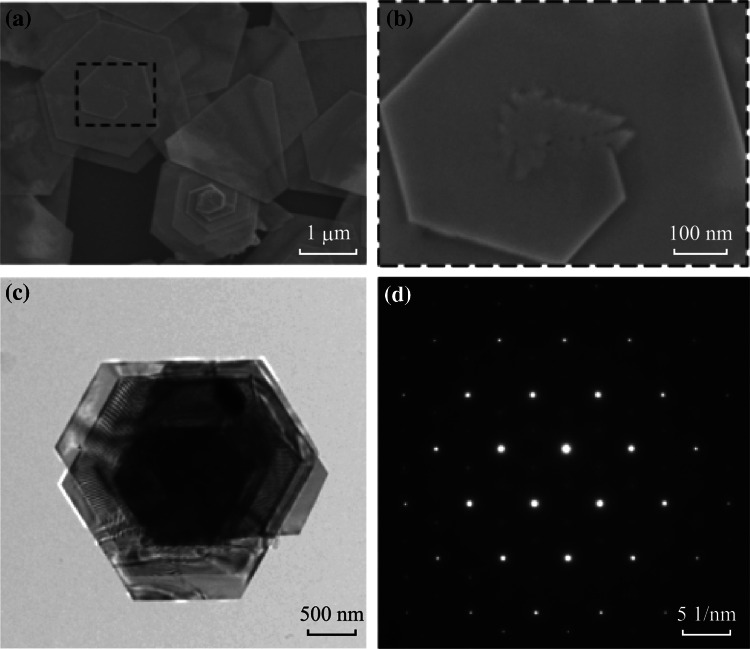
**a** SEM image of representative nanoplates. **b** The *zoomed up* image of the nanoplate core. **c** TEM, and **d** the corresponding SAED images

Figure 4 presents large nanoplates with several layers. The centers of the multi-layered spiral nanoplates in Fig. 4a have a stack of irregular gold nanostructures, due to the supply of gold atoms in the growth process. Figure S3 shows that the gold nanoparticles integrated with gold nanoplates have a tendency to be linearized, while other gold nanoparticles change from sphere-like nanoparticles to irregular polyhedron ones with crystal planes of lower energy exposed. TEM image in Fig. 4c of multi-layered spiral gold nanoplate shows Moiré fringes, indicating the orientation mismatches between different layers [50]. However, the orientation mismatches caused by dislocations or cracks between different lamellas are too tiny to be detected in the corresponding SAED patterns. The SAED image shows a typical super lattice patterns, implying that the multi-layered nanoplates are well crystallized and grow in a critical growth periodicity.

**Fig. 5:**
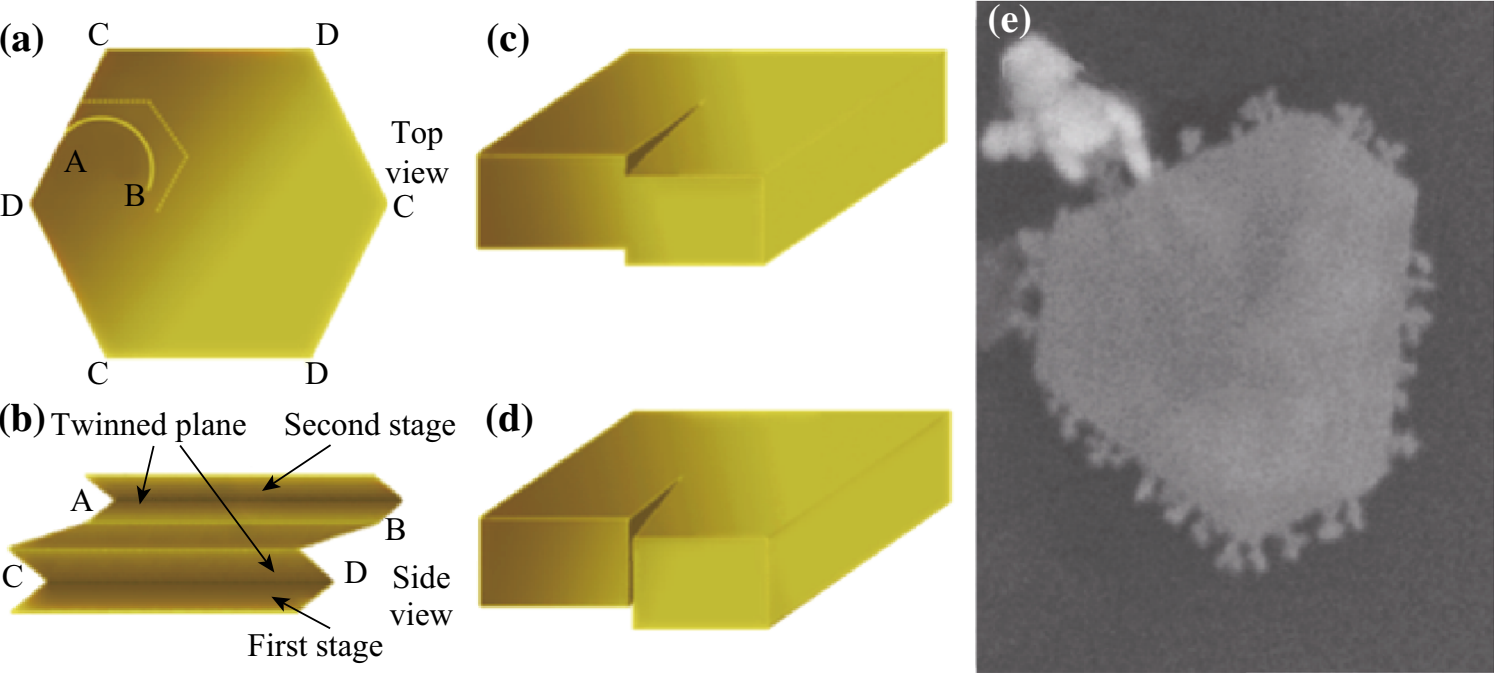
Schematic illustration **a** top view, and **b** side view of stage-induced spiral nanoplate. Two kinds of the stage origin: **c** dislocation and **d** tension crack. **e** SEM image of the small particles attaching at the edge of the nanoplate, as a proof of the edge-based growth process

**Fig. 6:**
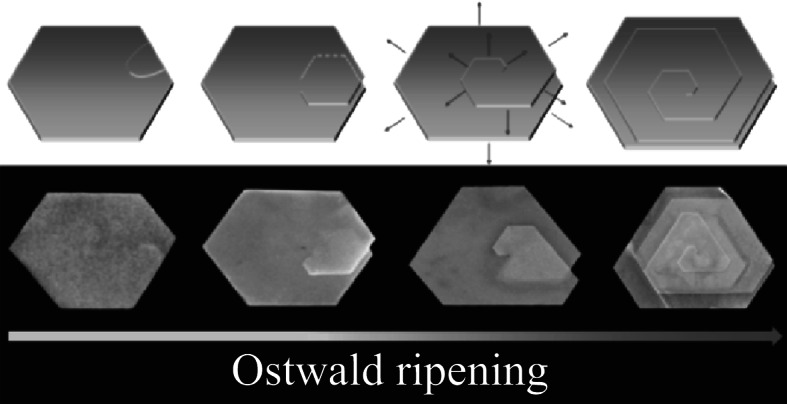
Schematic illustration of growth of the multi-layered spiral nanoplate (the *upper row*) and the corresponding SEM images (the *lower row*). From *left* to *right* are morphologies of different growth stages and gradually matured. *Red arrows* stand for the growth direction

Figure 5 shows the spiral nanoplates with several layers growing along edges. In the restructuring of gold atoms, multi-layered growth may be generated in two ways: one is caused by dislocations (Fig. 5c); the other owes to high interface tension cracks (Fig. 5d) [51]. When the sub-boundary stretches to surface or particles stack at the edge, dislocations are generated on the edge (Fig. 5e) [41]. Evidence of nanoplate cracks due to the excessive surface tension is shown in Fig. S4. The schematic stage-based growth routine is given in Fig. 6, corresponding to SEM images of spiral nanoplates at different ripening stage. The evolution belongs to an Ostwald ripening procedure to reduce the system energy. At the beginning, stages are generated due to high surface tension crack or nucleated dislocations by false stacking or sub-boundary stretching. Subsequently, the curved new edge is linearized to polygonal line parallel to the native edges [49]. All the layers of the gold nanoplates are growing, of which the growth direction is pointed by red arrows. Eventually, the monolayer nanoplates with larger sizes and layer numbers are formed.

### Possible Mechanism for the Biosynthesis of Gold Nanoplates

**Fig. 7:**
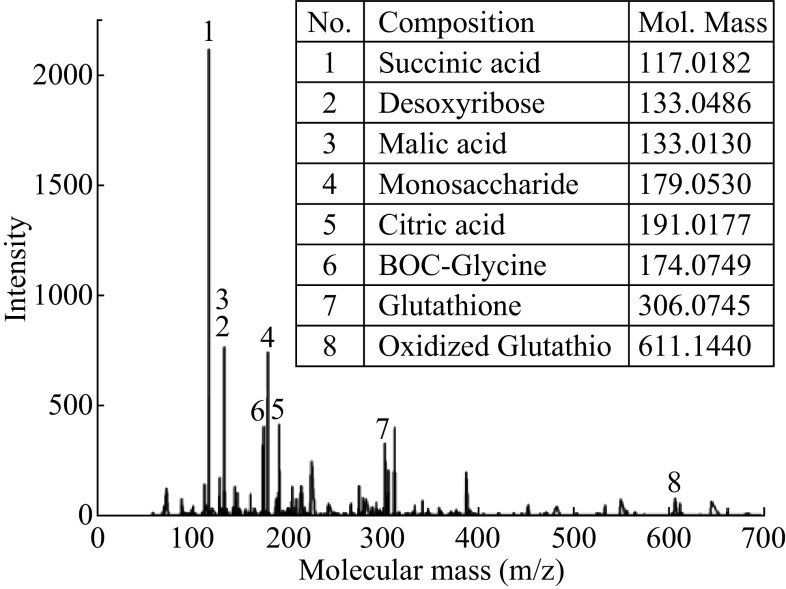
LC-ESI/HRMS spectrum of biomedium. The insert column lists specific compositions that may contribute to the biosynthesis of gold nanoplates. For molecules are tested in the negative ion mode (M–H), the measured molecule mass in the insert table is equal to the exact mass deducted which is the weight of 1 hydrogen atom

In order to detect the reactive species in the synthesis of gold nanoparticles, both the composition and the microscopic process in the reaction solution have been studied with sodium dodecyl sulfate-polyacrylamide gel electrophoresis (SDS-PAGE) and amino acid analysis (AAA) measurement. As shown in Fig. S5, no protein was detected in the fermentation liquor in the synthesis of gold nanoparticles. So proteins hardly make any contributions in the synthesis progress. Table S1 displays the other species, which mainly are multi-amino acid. It can be seen that glutamic acid (Glu) has the highest concentration. To identify the compositions more comprehensively, the fermentation liquor goes through high-performance liquid chromatography–electrospray ionizer/high resolution mass spectrometer (LC-ESI/HRMS). Succinic acid, lactic acid, and malic acid, which show high concentrations in the LC-ESI/HRMS, are products of Kreb’s cycle, providing the culture medium with a low pH (Fig. 7). Glutathione (GSH) with amino groups is of high concentration which plays an important role in preventing cells from dying and being oxidized under harsh conditions. All the species in the biosynthesis medium are organic molecules generated in metabolism induced by sucrose-only medium. In addition, the nutrient-induced condition can trigger the cell autolysis, reducing the effect of specific expression and improve the repeatability. It is also worth mentioning that, although the specific expression can be interrupted by the slight change of the surroundings, the autolysates of the cell are alike, ensuring that the experiment be repeated easily.

**Fig. 8:**
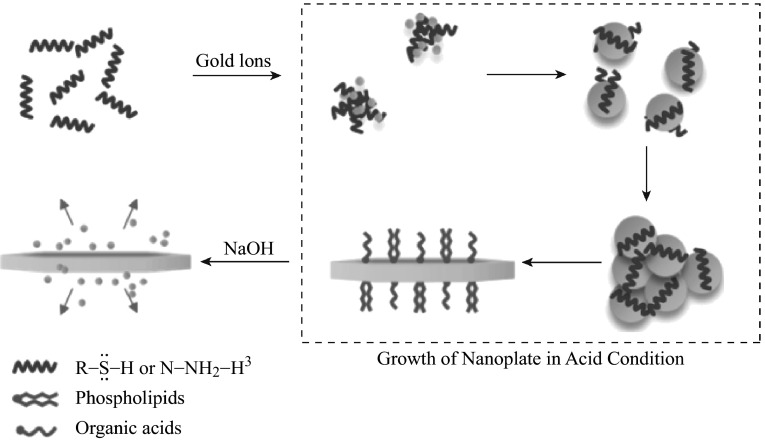
Schematic illustration of the growth of nanoplate, the gold ions have an evolution of nanoclusters, nanoparticles, and nanoplates, while the reverse reaction will occur when changing the pH of the solution later

Based on the analysis of the compositions of the biomedium, a schematic illustration of the synthesis approach is demonstrated in Fig. 8. Before the addition of HAuCl_4_, metabolites with thiol and amino groups are dispersed in the culture medium. As amino groups have a strong tendency to be protonated in the acid condition, molecules with these groups are positively charged. When the negative auric chloride acid complexes are introduced into the reaction system, the gold ions are entrapped by the biomolecules. It was observed in the reaction that once the two kinds of transparent reaction solutions were mixed together, the mixture got turbid and light colored [52]. There are still some metabolites with stronger reducibility in the reaction system, such as reductive sugar and sodium citrate, further reducing Au(I) to Au(0). In addition, ionized metabolites with a long chain, such as some phospholipid [53] and organic acids [54] can serve as surfactants. With the addition of NaOH, molecules with amino and thiol groups will become anionic and such anions are strongly nucleophilic, hence attacking and reducing Au(III) to form Au(I) coordination complex. Thus, there are more sites for nucleation and the final size of the product becomes smaller [55–57]. Besides, when gold nanoplates are re-dispersed in the base condition, gold nanoclusters with fluorescence are acquired (Fig. S6).

**Fig. 9:**
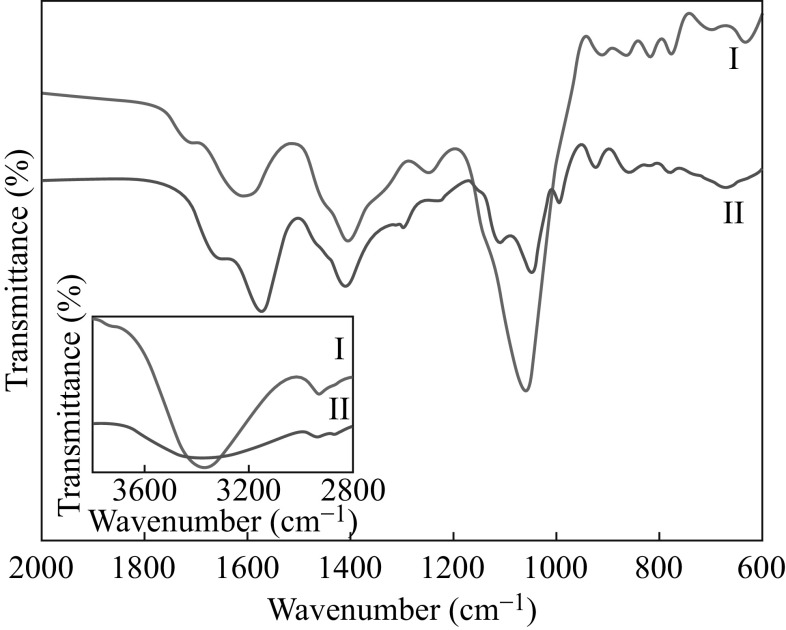
FT-IR spectra of yeast extract before (spectrum I) and after reaction (spectrum II) in 2000–600 cm^−1^, the *inset* shows the extending spectrum in 4000–2800 cm^−1^

FT-IR spectrum plays a key role in probing organics and analyzing the reaction phenomenon. In Fig. 9 and Table S2, the band at 1407 cm^−1^, and the three typical bands at 1703, 2931, and 3377 cm^−1^ of curve I are assigned to the amide I (mainly C=O stretching) and amide II (mainly N–H stretching) absorption due to C=O and N–H stretching vibrations, respectively. In general, amide I and amide II bonds are sensitive to the change of the accepting and donating group of adjacent parts. In spectrum II, the blue shift of the band from 1705 to 1654 cm^−1^ and the red shift of the band from 2931 to 2939 cm^−1^ imply that gold has a certain effect on the amine in the culture medium. Moreover, compared with the peaks at 674, 1064, 1604, and 3377 cm^−1^ in spectrum I, peaks in spectrum II at 682, 1069, 1571, and 3377 cm^−1^ weaken sharply, suggesting that the corresponding bond, C–N (amine), plays an important role in the bio-reduction of AuCl_4_^−1^. Besides, most of the bonds have shifts such as from 674 to 682, 1064 to 1049, and 1604 to 1571 cm^−1^. This suggests the mutual effect between gold nanoplates and the bio-reductants.

### Plasmonic Properties of Gold Nanoplates

In order to reveal the plasmonic properties, triangular, truncated triangular, and hexagonal nanoplates with the same thickness (15 nm) are chosen for FDTD calculation. The triangular nanoplate is modeled as a triangle with three edges of equal length of 400 nm. For the truncated triangular nanoplates, the length of the truncated edge is set as 80 and 150 nm, respectively. The edge length of the hexagonal nanoplate is 200 nm. The length between the opposite apexe, 400 nm, is the same for the four shapes of the nanoplates so that the nanoplates are kept at a similar size.

**Fig. 10:**
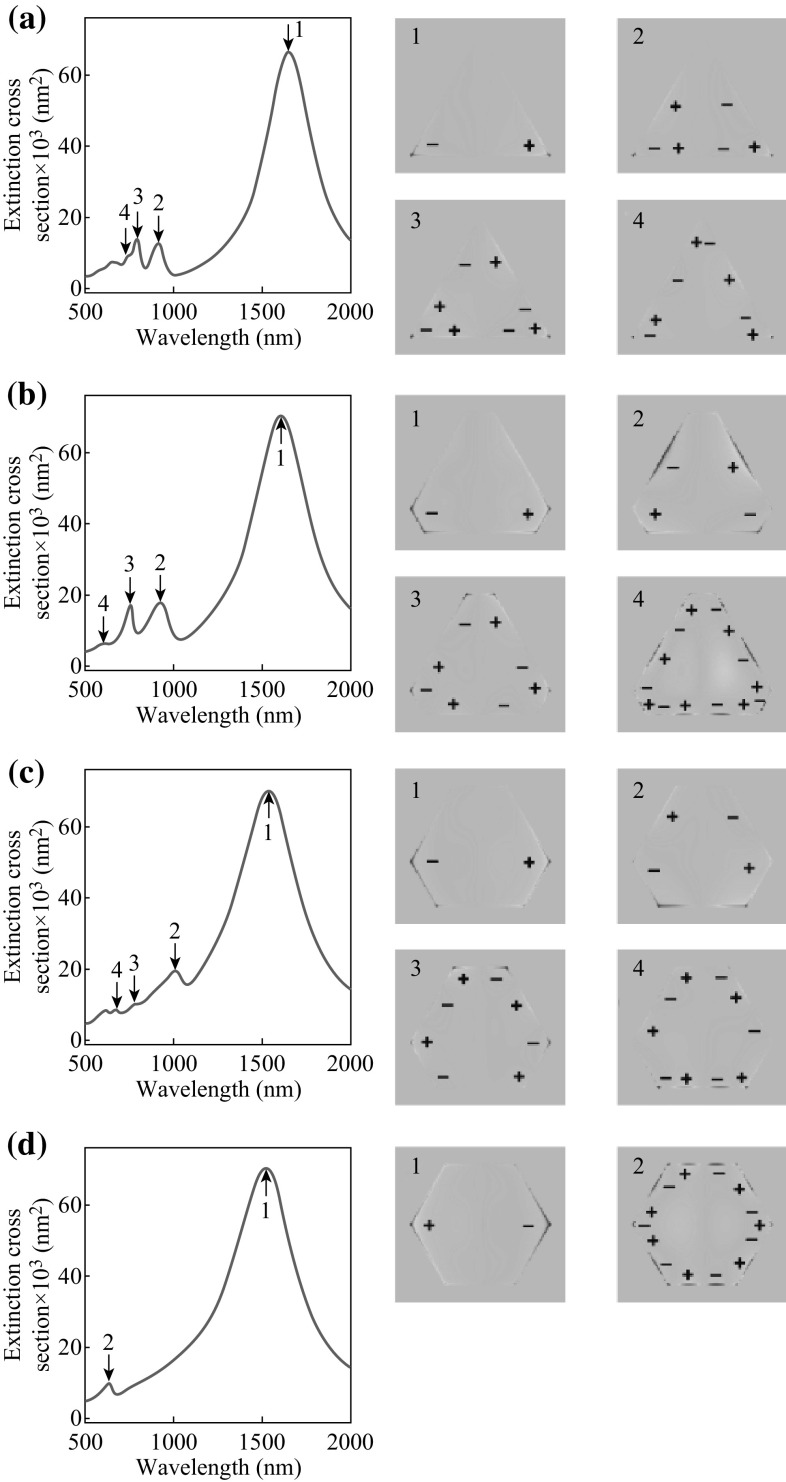
**a** Calculated extinction spectra (*left*) and corresponding charge distribution profiles (*right*) of the triangular gold nanoplate with 400 nm edges. Four plasmonic resonance modes can be identified in the extinction spectra (*arrows*), corresponding to the charge distribution profiles of the dipole (*1*), quadrupole (*2*), and higher-ordered (*3*, *4*) modes. **b** Those for the truncated triangular nanoplate with 80 nm truncated edges. **c** Those for the truncated triangular nanoplate with 150 nm truncated edges. **d** Those for the hexagonal nanoplate with 200 nm edges

Figure 10 shows the calculated results. For the four gold nanoplates, a common prominent peak is found at about 1600 nm in the extinction spectra, revealing the dipole oscillation mode of the nanoplates. As the nanoplates are 15 nm in thickness and 400 nm in length, the aspect ratio is approximately 26, which determines that the dipole resonances are located in the infrared region. Compared with recent report about gold nanoplates synthesized by chemical method, the gold nanoplates show in-plane dipolar plasmon peak with red shift, which is in agreement with the previous finding that the aspect ratio increase of gold nanoplates leads to red shifts in the in-plane dipolar plasmon peak [51]. It can be clearly observed in the charge distribution profiles (Fig. 10, right panels) that the dipole modes are associated with the charge oscillation which mainly belongs to the opposite apexes of the nanoplates. In the near-infrared and visible region, several additional minor peaks can be identified in the extinction spectrum, which are attributed to quadrupolar and higher-ordered oscillation modes. These modes can be clearly revealed by the charge distribution profiles at their corresponding peak wavelengths. It is usually found that the intensity of an oscillation mode decreases with its order. Moreover, in contrast to the dipole mode, the high-ordered oscillation modes are found to be very sensitive to the shape evolution of the nanoplate. When the shape performs an evolution from triangular to truncated triangular and finally to hexagonal nanoplates, intensities of the high-ordered modes gradually decrease. This suggests that along with the hexagonization of triangle nanoplates, the order of rotational symmetry of the nanoplate increases so that there is less room for the higher-ordered oscillation modes. Therefore, for the hexagonal nanoplate, only two modes, a dipole and a high-ordered one, can be found in the extinction spectra.

## Conclusions

One-pot biosynthesis of gold nanoplates with controllable morphology and size has been demonstrated by bioreducing of HAuCl_4_ in yeast metabolism. Gold nanoplates can be obtained under acid conditions, while nanoflowers and nanoparticles were obtained under basic condition. It is interestingly found that, after being re-dispersed and maintained in the deionized water, nanoplates change their structures and morphologies to multi-layered spiral nanoplates. When the nanoplates were re-dispersed in NaOH solution, they were partially dissolved with generating photoluminescent gold nanoclusters. By detecting the composition of the nutrient-induced biomedium, it demonstrates that thiol and amino groups with the reversible reaction in the acid and basic condition play the key role of protonation of the organic groups in the biosynthesis of nanoplates. Besides, FDTD simulation shows that the gold nanoplates have prominent extinction spectra peak at about 1600 nm, demonstrating great potential in surface plasmon-enhanced applications or the infrared thermotherapy. This work is expected as an example in point to overcome problems of traditional chemical synthesis and pioneer a new way of controllable biosynthesis depending on fermentation industry.

## Electronic supplementary material

Below is the link to the electronic supplementary material.

Supplementary material 1 (PDF 458 kb)
