# Supplementary material for: Controllable Biosynthesis and Properties of Gold Nanoplates Using Yeast Extract
Source: Nanomicro Lett. 2016 Sep 13;9(1):5. doi: 10.1007/s40820-016-0102-8 (PMC6223771; doi:10.1007/s40820-016-0102-8)
Supplement: Supplementary file 1 — Supplementary material 1 (PDF 458 kb) [file 40820_2016_102_MOESM1_ESM.pdf]

Supporting Information for

Controllable Biosynthesis and Properties of Gold Nanoplates Using Yeast Extract

Zhi Yang[1,*], Zhaohui Li[1], Xuxing Lu[2], Fengjiao He[1], Xingzhong Zhu[1], Yujie Ma[1], Rong He[1], Feng Gao[1], Weihai Ni[2,*], Yasha Yi[3,*]

[1]Key Laboratory for Thin Film and Microfabrication of Ministry of Education, Department of Micro/Nano Electronics, School of Electronic Information and Electrical Engineering, Shanghai Jiao Tong University, Shanghai 200240, People's Republic of China

[2]Division of i-Lab, Key Laboratory of Nano-Bio Interface & Collaborative Innovation Center of Suzhou Nano Science and Technology, Suzhou Institute of Nano-Tech and Nano-Bionics, Chinese Academy of Sciences, Suzhou, Jiangsu 215123, People's Republic of China

[3]Integrated Nano Optoelectronics Laboratory, Department of Electrical and Computer Engineering, University of Michigan, Dearborn, MI 48128, USA & Energy Institute, University of Michigan, Ann Arbor, MI 48109

*Corresponding authors. E-mail: zhiyang@sjtu.edu.cn, whni2012@sinano.ac.cn, yashayi@umich.edu

Tel.: +86-21-34206398, Fax: +86-21-34205665

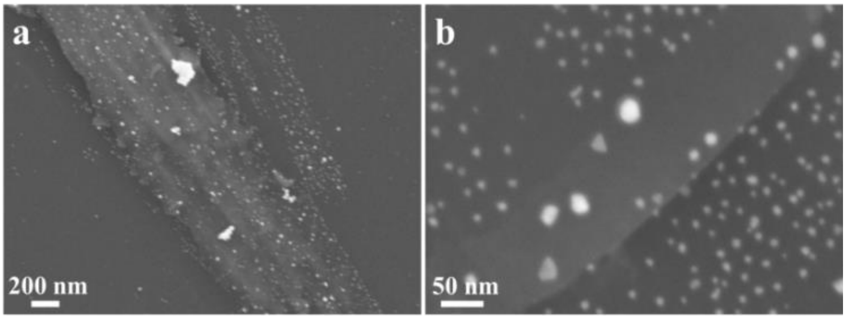

Fig. S1 At the first stage of the synthesis in the acid condition, only small nanoparticles were synthesized, without any nanoparticles.

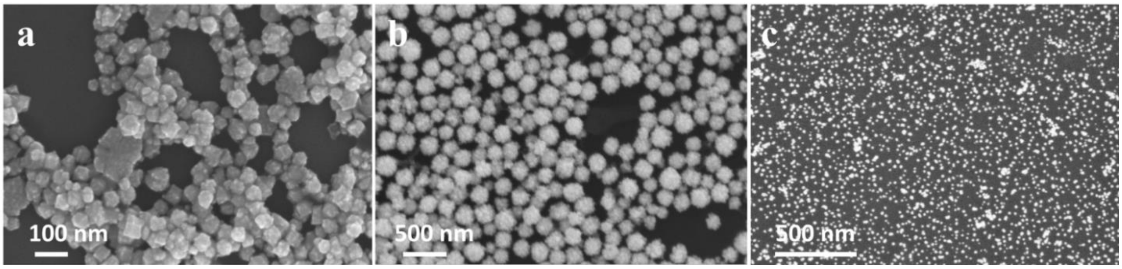

Fig. S2 SEM images of gold nanostructures synthesized at the pH values of 5.0 (**a**), 7.0 (**b**), and 10.0 (**c**). A mixture of plates and nanoflowers is acquired under the acid condition. In the neutral and base condition, the products come out to be nanoflowers and nanoparticles, respectively.

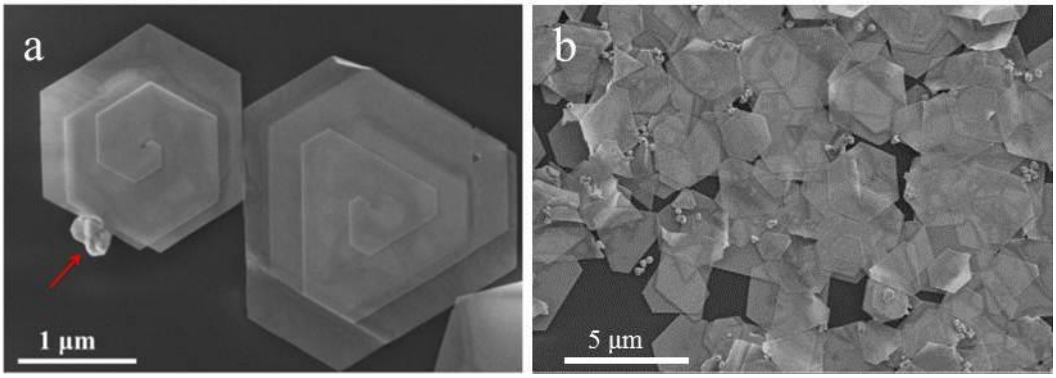

Fig. S3 SEM images show nanoparticles change their morphology along with the growth of spiral plates, which grow together and expose crystal planes of low interface energy.

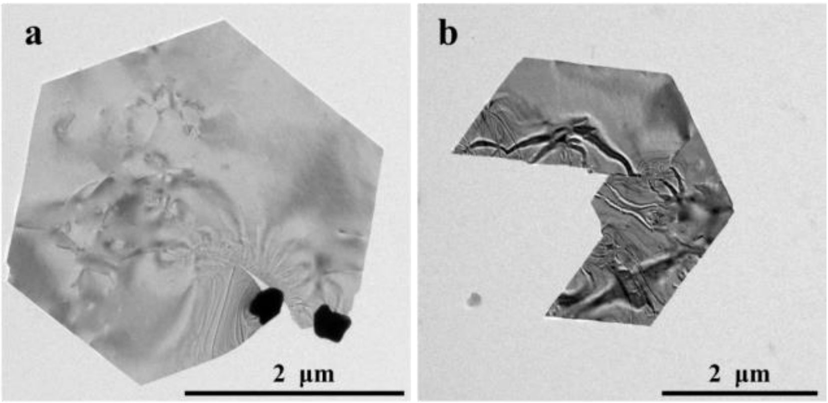

Fig. S4 TEM images of gold plates cracked, as a support of high surface tension.

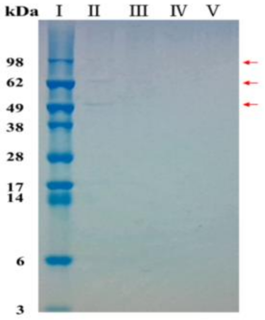

Fig. S5 Pre-stained protein standards sodium dodecyl sulfate polyacrylamide gel electrophoresis (SDS-PAGE) analysis of yeast medium extract of different second step concentration and heat process; with a second step centrifugation at 3000 rpm (line II), 8000 rpm (line III); with a second step centrifugation at 3000 rpm (line IV) or 8000 rpm (line V), 0.5 h's boiling and the third step of centrifugation at 8000 rpm. Line I shows standard protein molecular weight markers with molecular weights in kDa. At the first low speed step, yeast cells were precipitated and prevented form cell rupture, while at the second high speed step, hypha and cell debris were centrifuged as sedimentation.

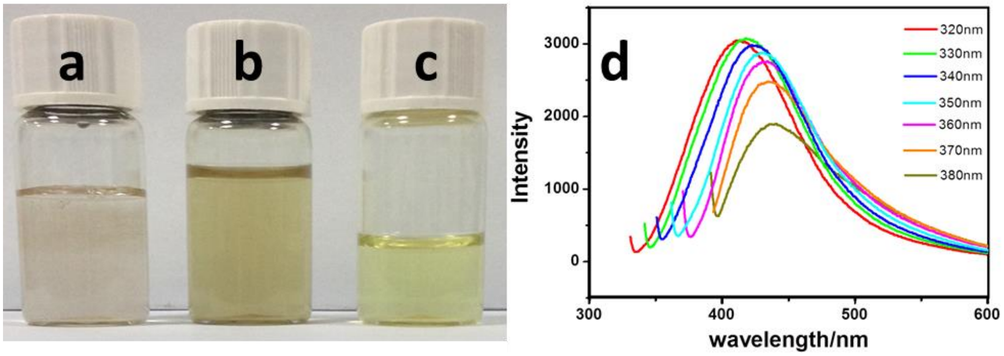

Fig. S6 Photo of plates in deionized water (**a**), plates re-dispersed in NaOH (**b**), and gold nanoclusters in base condition (**c**). Gold nanocluster solution was the supernatant of solution b going through a centrifugation at 10,000 rpm for 10 min; the corresponding photoluminescence spectra of nanoclusters recorded for progressively longer excitation wavelengths from 320 to 380 nm in a 10 nm increment (**d**).

Table S1 Amino acid analysis (AAA) of yeast medium abstract. 25 kinds of amino acids are detected, among which glutamic acid (Glu), amino n-butyric acid (a-ABA), and asparagine ($AspNH_2$) are the most ones. Especially, the glutamic acid, as the nitrogen source, has the concentration an order of magnitude higher than other materials.

Name	Area	Resolution	Mol concentration (nmol mL^{-1})	CalcMol (ng mL^{-1})
Asp	345417	1.41	9.392	1250.067
Thr	29462	6.51	0.763	90.839
Ser	52056	1.84	1.36	142.897
$AspNH_2$	27704	3.33	2.034	268.637
Glu	857271	1.68	20.706	3045.919
$GluNH_2$	46327	1.38	1.858	271.621
Gly	82343	13.91	2.073	155.648
Ala	521032	1.88	13.293	1184.434
Val	120456	6.54	3.058	358.062
g-ABA	183718	29.27	4.804	495.293
Hylys	14688	27.36	0.422	68.462
Orn	61505	7	1.445	190.984
Arg	63316	20.71	1.782	310.458

Table S2 Peaks changed in the FT-IR spectrum of yeast medium extract before and after the bioreduction. Weakened peaks means the corresponding bonds becomes less; strengthened peaks means the corresponding bonds accumulate in the redox reaction.

Peaks (before reaction)	Peaks (after reaction)	Strengthen or Weaken	Corresponding bonds	Chemical Compound
674	682	Weaken	N-H	Amine
1064	1069	Severely weaken	N-H	Amine
1407	1407	Weaken	N-H	Amine
1604	1571	Strengthen	N-H	Free amino acid
2931	2950	Weaken	N-H	Free amino acid
3377	3377	Weaken	N-H or O-H	Amine or sugar
